# Effect of antiretroviral therapy on longitudinal lung function trends in older children and adolescents with HIV-infection

**DOI:** 10.1371/journal.pone.0213556

**Published:** 2019-03-21

**Authors:** Sarah Rylance, Jamie Rylance, Grace McHugh, Edith Majonga, Tsitsi Bandason, Hilda Mujuru, Kusum Nathoo, Sarah Rowland-Jones, Marc Y. R. Henrion, Victoria Simms, Rashida A. Ferrand

**Affiliations:** 1 Liverpool School of Tropical Medicine, Liverpool, United Kingdom; 2 Malawi-Liverpool-Wellcome Trust Clinical Research Programme, Blantyre, Malawi; 3 Biomedical Research and Training Institute, Harare, Zimbabwe; 4 London School of Hygiene and Tropical Medicine, London, United Kingdom; 5 University of Zimbabwe, Harare, Zimbabwe; 6 University of Oxford, Oxford, United Kingdom; UKBB Universitats-Kinderspital, SWITZERLAND

## Abstract

**Introduction:**

Chronic respiratory disease is a common cause of morbidity in children with HIV infection. We investigated longitudinal lung function trends among HIV-infected children, to describe the evolution of lung disease and assess the effect of anti-retroviral therapy (ART).

**Methods:**

Prospective follow-up of two cohorts of HIV-infected children, aged 6 to 16 years, in Harare, Zimbabwe; one group were ART-naïve at enrolment, the other established on ART for a median of 4.7-years. Standardised spirometric assessments were repeated over a 2-year follow-up period. Forced expiratory volume (FEV_1_) and forced vital capacity (FVC) were expressed as Global Lung Initiative defined z-scores (FEV_1_z and FVCz). Linear mixed-effects regression modelling of lung function was performed, with co-variate parameters evaluated by likelihood ratio comparison.

**Results:**

We included 271 ART-naïve and 197 ART-established children (median age 11 years in both groups) incorporating 1144 spirometric assessments. Changes in FEV_1_ and FVC were associated with age at ART initiation and body mass index for both cohorts. Our models estimate that ART initiation earlier in life could prevent a deterioration of 0.04 FVCz/year. In the ART-naïve cohort, likelihood ratio comparison suggested an improvement in 0.09 FVCz/year during the two years following treatment initiation, but no evidence for this among participants established on ART.

**Conclusion:**

Early ART initiation and improved nutrition are positively associated with lung function and are important modifiable factors. An initial improvement in lung growth was seen in the first 2-years following ART initiation, although this did not appear to be sustained beyond this timeframe.

## Introduction

Increasing numbers of children with HIV, who would previously have died in early infancy, are now reaching adolescence due to the remarkable global scale-up of paediatric antiretroviral therapy (ART).[1] In addition, one-third of HIV-infected infants in Sub-Saharan Africa, where 90% of the world’s HIV-infected children live, have slow-progressing disease with a median survival of more than a decade, even without ART.[2, 3]

In recent years, several studies from sub-Saharan Africa have demonstrated a high prevalence of chronic respiratory symptoms in older children and adolescents with HIV, including dyspnoea, hypoxia at rest and during sub-maximal exercise.[4] In studies conducted in Zimbabwe, on which data this paper is based, 28% of ART-naïve and 24% of ART-experienced children aged 6 to 16 years had abnormal lung function, most frequently reduced forced vital capacity (FVC).[5, 6]

Cross-sectional data from a Danish cohort of well treated HIV-infected adults found decreased FEV and FVC, compared to matched HIV-noninfected controls, with decreased lung function associated with CD4 nadir.[7] Longitudinal analyses of lung function in HIV-infected adults in high-income settings have demonstrated a decline in lung function associated with poorly controlled HIV infection and acute lung infection. However, studies are confounded by high rates of smoking and drug use.[8, 9] A substudy to explore lung function within the Strategic Timing of Antiretroviral Treatment (START) trial, found no difference in FEV1 decline over a median two-year period, between those receiving immediate or delayed ART.[10] However, there are no comparable data for the paediatric population.

The existing cross-sectional studies provide limited description of the evolution of chronic lung disease in African children. In particular, the effect of antiretroviral therapy and timing of ART initiation on lung function is unclear. This is important as many children in Africa are identified with HIV and started on ART only in later childhood and adolescence, and longstanding uncontrolled HIV infection may be associated with worsening lung function.[3] We hypothesised that initiation of ART earlier in life would be advantageous to lung function.

We present the longitudinal lung function results from two cohorts of children aged 6–16 years living with HIV, one with recently diagnosed HIV and the other established on ART.[5, 6]

## Methods

Detailed methods have been previously published for both cohorts.[5, 6] Children were eligible if aged 6–16 years, were not acutely unwell (no acute symptoms and not requiring hospitalisation), and were not receiving TB treatment.

Briefly, participants who were on ART for at least six months were recruited from a public sector HIV clinic at Harare Hospital, in Zimbabwe, between September 2014 and June 2015. Data were collected at baseline and a follow-up spirometry assessment was performed at 18-months. Participants in the ART- naïve cohort were recruited if they tested HIV-positive through provider-initiated HIV testing and counselling, at seven public sector primary healthcare clinics in Harare (serving the same catchment population as Harare Hospital) between January 2013 and December 2014. Participants underwent spirometry six-monthly over a two-year period. HIV infection was treated according to the Zimbabwe national guidelines: prior to February 2014 children aged over five-years were started on ART if their CD4 count was below 350 cells/mm^3^ or if they had WHO stage 3 or 4 HIV disease. After March 2014, Zimbabwe adopted the WHO 2013 guidelines, with a revised threshold for ART initiation of 500 cells/mm.^3^[11] We therefore excluded those not immediately started on ART from this analysis.

Written informed consent was obtained from all caregivers and written assent from participants. Ethical approval was granted by the Medical Research Council of Zimbabwe, the Harare City Health Department Ethics Committee, the London School of Hygiene and Tropical Medicine Ethics Committee and the Biomedical Research and Training Institute Institutional Review Board.

### Data collection

Details of socio-demographic indices, clinical history and current symptoms were collected through a nurse-administered questionnaire. Data was extracted from paper forms using Cardiff TELEFORM Intelligent Character Optical Mark Recognition Software (Version 10.9; Hewlett Packard, California, USA). Spirometry was performed according to American Thoracic Society (ATS) standards using an EasyOne World Spirometer (NDD Medical Technologies, Inc., Andover, Massachusetts, USA). Forced exhalations were recorded while sitting, with repeated attempts until quality criteria had been reached or eight attempts completed. Data were included for analysis if individuals produced at least 2 spirometry traces without artefact, with reproducible highest forced expiratory volume in 1s (FEV_1_) and forced vital capacity (FVC) values (traces within 100ml or 10% of each other), in accordance with ATS quality criteria.[12] An absolute time value was not used for duration of test, but expiratory curves must have reached a plateau in each usable trace.

### Statistical analysis

The FEV_1_ and FVC measurements for each participant were expressed as a z-score (FEVz and FVCz), using normal spirometric ranges defined by the Global Lung Initiative equation which provides race- and sex-specific reference values, accounting for height and age.[13] Z-scores describe the variation from the expected value according to the reference range (1z score is one population standard deviation. A value of 0 represents the expected value, with negative values below expected).

The relationship between FVCz and FEVz and explanatory co-variates (time on ART, age at ART initiation, and BMI z-score) was investigated by linear mixed-effects regression modelling of longitudinal data, using the *lme4* package within R (v3.3.3, the R project).[14, 15] A mixed-effects model describes the population-average (fixed) effect and subject-specific (random) effects, allowing for between-subject variability by introducing individual-specific intercepts and slopes over time.[16] The model assumes that the distribution of the random effects does not depend on the values of any explanatory variables included in the model. The full fixed effects model is described below:

$$Y_{ij}=\beta_{0}+\beta_{1}∙t_{ij}+\beta_{2}∙{a_{i}}^{\left(0\right)}+\beta_{3}∙{a_{i}}^{\left(0\right)}∙t_{ij}+\beta_{4}∙b+U_{i}+V_{i}∙t_{ij}+Z_{ij}$$

where:

*Y*_*ij*_ = FEVz or FVCz response for patient *i* at visit *j*

*t*_*ij*_ = time (in years) since ART initiation for patient *i* at visit *j*

*a*_*i*_^(0)^ = age (in years) at ART initiation for patient *i*

b = BMI z-score for patient *i*

*β*_0_, *β*_1_, *β*_2_, *β*_3_, *β*_4_ = fixed effects parameters

(*U*_*i*_, *V*_*i*_) ~ *N*(0, *D*), random effects associated with patient *i*

$$D=\left[\begin{array}{cc} \sigma_{u}^{2} & \sigma_{uv}\\ \sigma_{uv} & \sigma_{v}^{2} \end{array}\right]$$

$$Z_{ij}\sim N(0,{\sigma_{x}}^{2})$$

The full model was fitted to the data, with evaluation of different random effects; 1) no random effects, 2) individual intercept, and 3) individual intercept and slope. Maximized log-likelihood values were compared for parameters in nested models, using restricted maximum likelihood (REML) estimation and parameters carried forward if their inclusion resulted in a significantly improved model fit as assessed by likelihood ratio testing. Fixed effects parameters were then sequentially evaluated using maximum likelihood (ML) estimation, with likelihood ratio comparison to select the final models and estimate parameter values. To verify the assumptions of a linear mixed model, Pearson residuals were plotted against the fitted values for the final lung function response models.

## Results

Data for 202 ART-experienced and 385 ART-naïve children were available; detailed baseline cohort characteristics have been published.[5, 6] Of the latter, 307 met criteria for, and were established on, ART during the follow-up period: 78 children did not receive ART during the study period, and were not included in the longitudinal data analysis, which was designed to assess the effect of ART on lung function. Table 1 summarises the baseline characteristics of both cohorts. Age and sex distributions were similar between groups (Table 1). As expected, ART-naïve participants were older at HIV diagnosis than those on treatment (median 11.0 years [IQR 9.0–13.0] vs. 4.9 years [IQR 2.8–7.5] respectively, p<0.001) and had significantly lower CD4 counts at enrolment (median 313 cells/μl [IQR 193–490] vs. 727 cells/μl [IQR 478–938], p<0.001).

**Table 1:** Comparison of ART-naïve and ART established cohorts at baseline.

	Initially ART naïve *n = 307*	Receiving ART *n = 202*	p value
Age, median years (IQR)	11.0 (9.0–13.0)	11.1 (9.0–12.9)	0.63
Male sex, n (%)	147 (48)	111 (55)	0.14
Age at diagnosis, median years (IQR)	11.0 (9.0–13.0)	4.9 (2.8–7.5)	<0.001
CD4 count at study enrolment, median cells/μl (IQR)	313 (193–490) ^a^	727 (478–938) ^b^	<0.001
Viral load <400 copies/ml at study enrolment, n (%)	Data not collected	155/197 (78.6)	-
Duration of ART at recruitment, median years (IQR)	0	4.7 (2.6–6.4)	-
FEV, mean z-score (SD)	-0.80 (1.31) ^c^	-0.75 (1.25) ^d^	0.66
FVC, mean z-score (SD)	-0.73 (1.29) ^c^	-0.81 (1.28) ^d^	0.55
HFA, mean z-score (SD)	-1.65 (1.18) ^c^	-1.61 (1.09) ^d^	0.69
BMI for age, mean z-score (SD)	-0.96 (1.23) ^c^	-0.53 (1.08) ^d^	<0.001

Abbreviations: ART, antiretroviral therapy; FEV, forced expiratory volume in 1 second; FVC, forced vital capacity; HFA, height for age; BMI, body mass index; IQR, interquartile range; SD, standard deviation

^a^ Baseline CD4 count available for 232 children

^b^ Data available for 200 children

^c^ Acceptable baseline spirometry and anthropometry for 271 children

^d^ Acceptable baseline spirometry and anthropometry for 177 children

**Fig 1:**
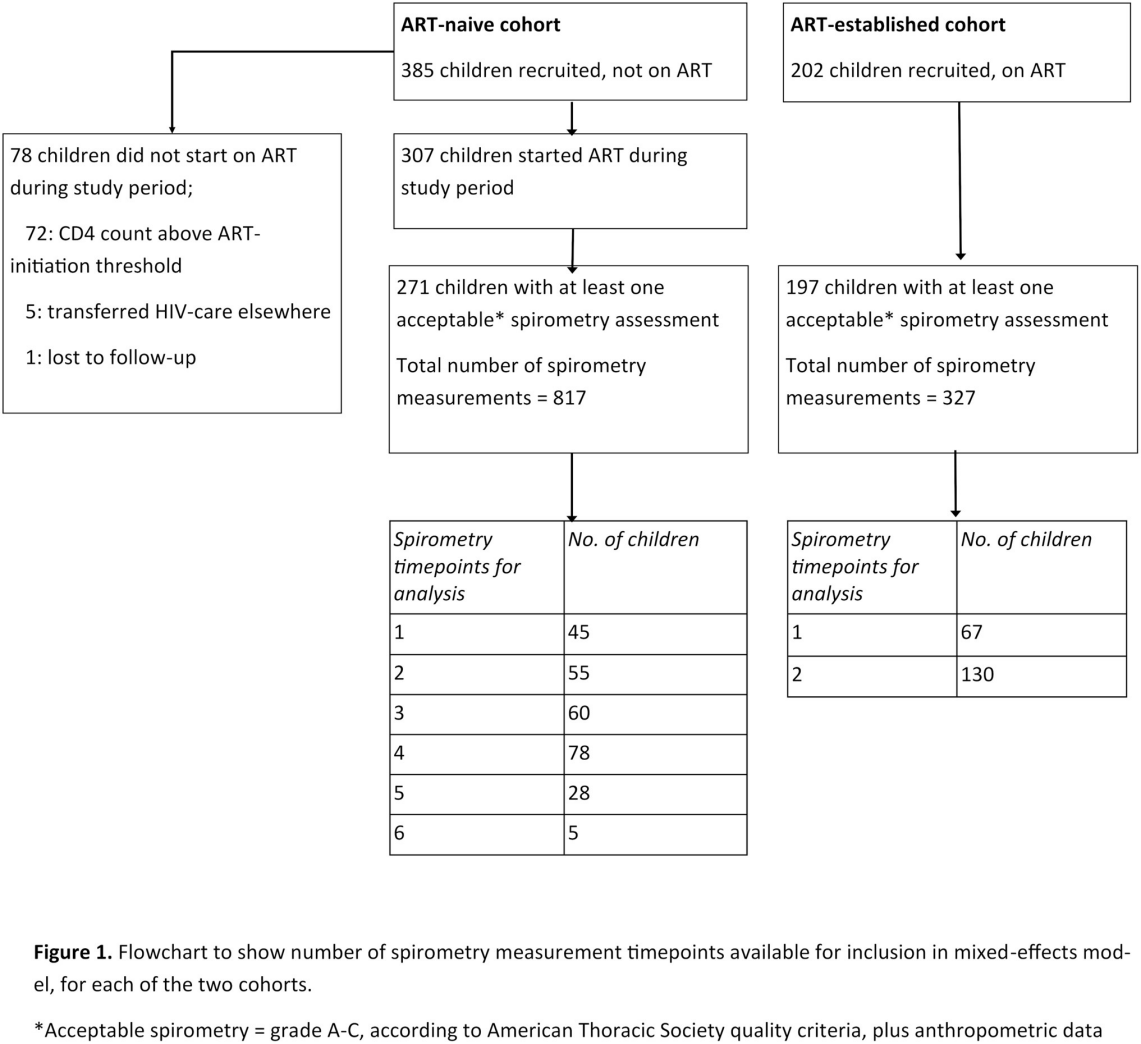
Flowchart to show number of spirometry measurement timepoints available for inclusion in mixed-effects model, for each of the two cohorts; ART naïve and ART established. *Acceptable spirometry = grade A-C, according to American Thoracic Society quality criteria, plus anthropometric data available.

For those with longitudinal data, the median duration of follow-up was 570 days for the ART-naive children and 523 days for the ART-established children. A total of 1144 spirometry assessments from 468 children met ATS quality criteria for inclusion in the analysis; 817 assessments from 271 ART-naïve children and 327 assessments from 197 ART-established children (Fig 1). The duration of ART at the time of spirometry assessment ranged from 0–14.3 years. Both cohorts had comparable baseline lung function, with mean FEV z score -0.8 (SD 1.31) and FVC z-score -0.73 (SD 1.29) for ART-naïve children, and mean FEV z-score -0.75 (SD 1.25) and FVC z-score -0.81 (SD 1.28) for those already established on ART.

For the ART-naïve cohort, comparison of nested models by maximized log-likelihood value selected the inclusion of individual intercept and slope as random effects (S1 and S3 Tables). We were unable to fit an identical model to the ART-established cohort dataset, due to fewer timepoints per individual, with only one timepoint for one-third of this cohort; this model therefore included an individual intercept only (S5 and S7 Tables). For the ART-naïve cohort, inclusion of time on ART, age at ART initiation and BMI z-score, as explanatory co-variates, significantly improved the FVC z-score model fit (p<0.05 for each) (S2 Table). Time on ART did not contribute significantly to the model for FEV z-score, in comparison to age at ART initiation and BMI z-score (S4 Table). Time on ART was also not included in the final FEVz and FVCz models for the ART-established cohort. However, age at ART initiation and BMI z-score significantly improved the model fit for both lung function measurements (S6 and S8 Tables). The interaction between age at ART initiation and time on ART was not included in the final model, for either cohort.

**Fig 2:**
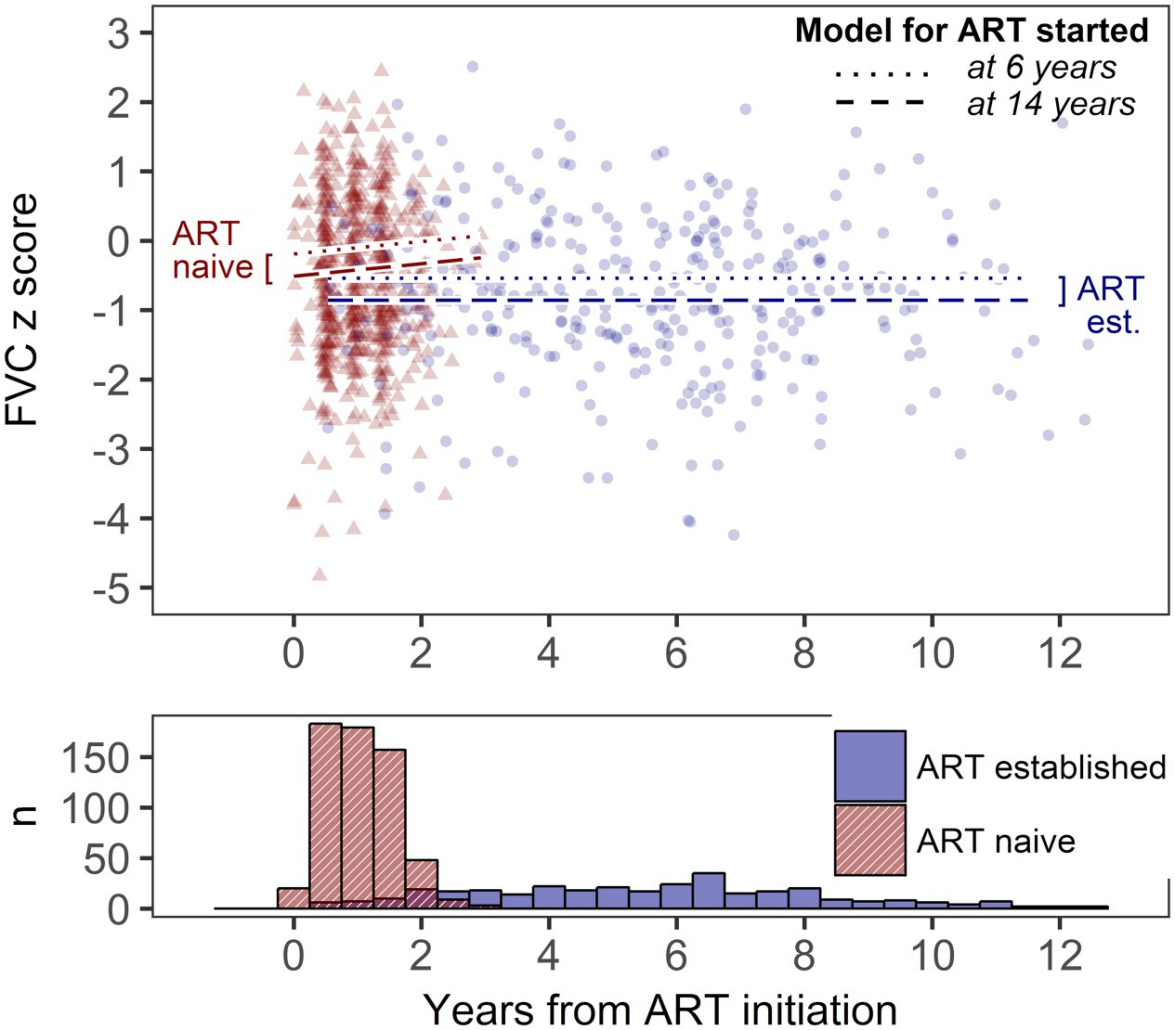
Models of FVC from original cohort data. Original spirometric data representing forced vital capacity (FVC) z-scores are shown for ART naïve participants (triangles) and ART established participants (circles). Both panels have equivalent x axes showing the time from ART initiation at which spirometry was performed. The lower panel (histogram) describes the number of measurements contributing to analysis in each 6-month interval. Superimposed on the upper panel are two pairs of lines representing the final statistical model. For each cohort two model scenarios are given: dashed lines for individuals starting ART at age 14 years, and dotted lines for individuals commencing ART age 6 years.

Table 2 shows a comparison of the final mixed-effects model parameter estimates, for the ART-naïve and ART-established cohorts. For the ART-naïve cohort, inclusion of time on ART significantly improved the model fit for FVC z-score. This equated to an increase in FVC z-score of 0.09/year on ART for a cohort-mean BMI z-score and age at ART. For both cohorts, age at ART initiation was negatively associated with FVC and FEV z-score. Fig 2 illustrates the differences in FVC z-score, associated with ART initiation at age 6 and 14 years, for children with average BMI z-score (a decrease in FVC z-score of 0.32 for the 8 years delay). For both ART-naïve and ART-established datasets, nutritional status (measured by BMI z-score[17]) at enrolment was significantly associated with FVC and FEV z-scores.

**Table 2:** Parameter estimates for final mixed-effects FVCz and FEVz response models for both ART-naïve and ART-established cohorts.

	ART-naïve		ART-established	
	FVC z-score	FEV z-score	FVC z-score	FEV z-score
	*Estimate (95%CI)*	*Estimate (95%CI)*	*Estimate (95%CI)*	*Estimate (95%CI)*
**Fixed effects**				
Intercept: β_0_	0.05 (-0.51, 0.62)	0.06 (-0.54, 0.67)	-0.30 (-0.61, 0.00)	-0.16 (-0.48, 0.16)
Time on ART: β_1_	0.09 (0.01, 0.18)	#	#	#
Age at ART initiation: β_2_	-0.04 (-0.09, 0.00)	-0.06 (-0.11, 0.00)	-0.04 (-0.09, 0.00)	-0.06 (-0.11, -0.02)
Time on ART*Age at ART initiation: β_3_	#	#	#	#
BMI z-score: β_4_	0.26 (0.20, 0.32)	0.18 (0.12, 0.24)	0.37 (0.24, 0.49)	0.31 (0.19, 0.43)
**Random effects** for each subject				
Intercept variance: σ_u_^2^	1.04	1.13	0.74	0.88
Time on ART slope variance: σ_v_^2^	0.11	0.10	#	#
Correlation: σ_v_^2^	-0.47	-0.34	-	-

CI confidence intervals

^#^: model not significantly improved by inclusion of this parameter, therefore omitted in final model

## Discussion

To our knowledge, this is the first published description of longitudinal lung function trends in HIV-infected children from Sub-Saharan Africa. Our data show that age at ART initiation and BMI both significantly explain variation in lung function (FEV_1_ and FVC). FVC improves over time in the 2-years following ART initiation, but this effect cannot be extrapolated beyond the period of the study.

Early childhood represents a crucial period of rapid lung growth, with exponential increase in alveolar numbers during the first 2-years of life and reduced growth velocity towards adolescence.[18],[19] In HIV-uninfected children aged below 5-years, pneumonia has been shown to be associated with long-term restrictive lung disease: parenchymal damage as a result of infection may reduce expansion and growth of alveoli, resulting in reduced vital capacity.[20, 21] In HIV-infected children, pulmonary viral and bacterial infections are much more frequent, resulting in an increased risk of chronic lung disease.[22] A study of HIV-infected children showed that a third had chronic chest radiographic changes, most commonly increased bronchovascular markings or reticular densities by 4 years of age.[23] Most had been treated with suboptimal ART (mono-or dual therapy). In a more recent study from Zimbabwe, altered lung architecture, evident on chest radiography as tramlines and ring opacities, was described in perinatally HIV-infected children diagnosed in adolescence.[24] In studies of older children and adolescents diagnosed with and treated for HIV beyond infancy, high resolution computed tomography studies have shown obliterative bronchiolitis (OB) as the most common cause of chronic lung disease.[25, 26] Airway inflammation due to infection (e.g adenovirus or mycoplasma) is a well-recognised risk factor for developing OB.[27]

ART reduces the risk of infections and reduces systemic immune activation associated with HIV infection.[28] In African and European children, ART improves short and long-term CD4 cell counts, suggesting that children achieve robust immune reconstitution.[29, 30] Maximising the immunological benefits through early ART initiation reduces the risk of respiratory infections and may have a beneficial impact on airways immune dysregulation, thus limiting the negative impact on lung architecture.[4, 31] Given that early childhood is a critical period for lung growth, it is likely that the positive effects of ART on the lung would be most pronounced in this period.[18] Our findings differ from the START substudy, which reported no difference between decline in FEV1 for adults receiving immediate or delayed ART: this may reflect the much younger age of our cohorts, who should still be achieving lung growth and development.[10] The most recent World Health Organization Guidelines recommend ART initiation in all individuals following HIV diagnosis regardless of disease or immunological stage, in contrast to previous guidelines which recommended immediate treatment only in children aged below two years.[11, 32] These recommendations are based on findings from clinical trials in adults demonstrating the benefits of early ART on mortality.[33, 34] Our findings strongly support the guideline shift to early universal ART in children in order to protect organ development during critical periods of growth and development.[32]

In our analysis, duration of ART was associated with change in FVC, but not significantly with FEV_1_, in children following ART initiation. We did not find this in the ART-established cohort, which may reflect insufficient longitudinal data to detect a significant difference, or could represent an initial response to ART, which subsides over time. The spirometric parameters measured describe different lung properties; FEV_1_, reflects airway calibre and elasticity, and FVC, lung volumes and growth.[35] During childhood and adolescence, differential rates of variation in both parameters have been observed.[36] The discrepancy between FVC and FEV_1_ response in the 2-years following ART initiation, could be explained by an initial positive impact on lung development, representing an opportunity for “catch-up” growth. Further longitudinal research, following large cohorts of children on ART could confirm this hypothesis, and demonstrate the critical time period for intervention.

Our finding that poor nutritional status (measured by BMI[37]) was significantly associated with lung function is supported by other studies of chronic respiratory disease, reporting a positive correlation between lung health and nutrition, for example cystic fibrosis.[38] In bronchopulmonary dysplasia in young children, above-average somatic growth, a proxy for nutrition, is associated with significant relative improvements in lung function.[39] Primary nutritional deficiency also limits somatic and lung growth, with proportional reductions in FEV_1_ and FVC seen in malnourished African school children (BMI z-score <-2).[40] While the nutritional status in our participants may partly reflect underlying immune-deficiency, supplementary dietary interventions, particularly among malnourished children may help to maximise lung growth and maturation.

The strengths of the study were that spirometry was conducted by experienced staff, according to ATS quality control guidelines to minimise systematic errors, with 88% in each cohort meeting these standards.[12] However, those unable to produce acceptable traces may include those with more severe lung disease. We acknowledge several limitations. In order to maximise the longitudinal lung function data available for analysis we have combined two cohort studies, and therefore measurements have been collected as part of different follow-up schedules. Clinical data collected were different for each cohort, and therefore we have restricted our analysis to explore lung function and anthropometry, rather than impute missing variables for a substantial number of participants, and have not corrected the model for multiple statistical testing. Further research should explore the impact of potential clinical modifiers on longitudinal lung function such as CD4 count, viral load, and previous significant respiratory infections including TB. In the ART-naïve cohort, spirometry data points from all 5 per-protocol visits were available for only 9% (28/307), with the majority providing 3 or more spirometry measurements. Bias may have been introduced if participants did not attend visits due to perceived good health, or severe disease, although an advantage of the modelling approach meant that individuals acted as their own controls. Noise within the spirometry data limits the predictive capacity of our models. Within-subject variation arises from measurement error and physiological day-to-day fluctuation.[41] In studies of healthy school children, two-thirds of participants had between-test variability of less than 0.5z (~6% predicted) and 95% of participants had variation of less than 1.2z (~13% predicted) over 1 year.[42] We estimated an improvement of 0.18 FVC z-score (2.5% of predicted FVC) for an ART- naïve child, with a cohort-mean BMI z-score, over the 2-years following ART initiation. FVC z-score decreases by 0.04, for every year that ART initiation is delayed. While this may not be clinically significant at an individual level, it may be at a population level.

## Conclusions

In summary, our study supports the WHO policy of immediate ART initiation in children.[32] The beneficial effect of early ART on lung function was a consistent finding across both cohorts, and highlights a time-limited opportunity to intervene to promote optimum lung growth and development. Our data suggest that nutritional interventions may further contribute to improved lung health, although addressing both immunologic recovery and nutrition will be required to maximise benefits.

## Supporting information

S1 Table Likelihood ratio comparison of random-effects FVCz response models incorporating; 1: Residual error, 2: Individual intercept, 3: Individual intercept and slope for the ART-naïve cohort. (DOCX)

S2 Table Likelihood ratio comparison of increasingly complex mixed-effects FVCz response models for the ART-naïve cohort. (DOCX)

S3 Table Likelihood ratio comparison of random-effects FEVz response models incorporating; 1: Residual error, 2: Individual intercept, 3: Individual intercept and slope for the ART-naïve cohort. (DOCX)

S4 Table Likelihood ratio comparison of increasingly complex mixed-effects FEVz response models for the ART-naïve cohort. (DOCX)

S5 Table Likelihood ratio comparison of random-effects FVCz response models incorporating; 1: Residual error, 2: Individual intercept, for the ART-established cohort. (DOCX)

S6 Table Likelihood ratio comparison of increasingly complex mixed-effects FVCz response models for the ART-established cohort. (DOCX)

S7 Table Likelihood ratio comparison of random-effects FEVz response models incorporating; 1: Residual error, 2: Individual intercept, for the ART-established cohort. (DOCX)

S8 Table Likelihood ratio comparison of increasingly complex mixed-effects FEVz response models for the ART-established cohort. (DOCX)

S1 File INHALE_clinical questionnaire. Questionnaire for ART-experienced cohort.
(PDF)

S2 File ZENITH_clinical questionnaire. Questionnaire for ART-naïve cohort.
(PDF)
