# Supplementary material for: Effect of antiretroviral therapy on longitudinal lung function trends in older children and adolescents with HIV-infection
Source: PLoS One. 2019 Mar 21;14(3):e0213556. doi: 10.1371/journal.pone.0213556 (PMC6428265; doi:10.1371/journal.pone.0213556)
Supplement: S1 Table — (DOCX) [file pone.0213556.s001.docx]

**Table S1.** Likelihood ratio comparison of random-effects FVCz response models incorporating;

**1**: Residual error, **2**: individual intercept, **3**: individual intercept and slope for the ART-naïve cohort.

|  | *Random effects* |  | *Comparison* | *LogLikelihood* | *Likelihood ratio test* | *p-value* |
| --- | --- | --- | --- | --- | --- | --- |
| **1** | Z_ij_ |  | - | -1228.7 | - | - |
| **2** | U_i_, Z_ij_ |  | 1 and 2 | -1000.3 | 456.7 | < 2.2e^-16^ |
| **3** | U_i_, V_ij_, Z_ij_ |  | 2 and 3 | -989.4 | 21.8 | 1.1 e^-05^ |
