# Supplementary material for: Effect of antiretroviral therapy on longitudinal lung function trends in older children and adolescents with HIV-infection
Source: PLoS One. 2019 Mar 21;14(3):e0213556. doi: 10.1371/journal.pone.0213556 (PMC6428265; doi:10.1371/journal.pone.0213556)
Supplement: S2 Table — (DOCX) [file pone.0213556.s002.docx]

**S2 Table.** Likelihood ratio comparison of increasingly complex mixed-effects FVCz response models for the ART-naïve cohort.

|  | *Fixed effects parameters* | *Comparison* | *LogLikelihood* | *Likelihood ratio test* | *df* | *p-value* |
| --- | --- | --- | --- | --- | --- | --- |
| **1** | β_0_ | - | -1017.9 | - | - | - |
| **2** | β_0_, β_1_ | 2 and 1 | -1014.0 | 7.8 | 1 | 0.005 |
| **3** | β_0_, β_1,_ β_2_ | 3 and 2 | -1011.6 | 4.9 | 1 | 0.028 |
| **4** | β_0_, β_1,_ β_2_, β_3_ | 4 and 3 | -1011.4 | 0.3 | 1 | 0.564 |
| **5** | β_0_, β_1,_ β_2_, β_4_ | 5 and 3 | -976.7 | 69.9 | 1 | < 2.2e^-16^ |

* All models have σ_u_^2^, σ_v_^2^_,_ σ_z_^2^, σ_uv_ as parameters. Parameters relate to; β_0_, intercept; β_1_, time on ART; β_2_, age at ART initiation; β_3_, interaction between time on ART and age at ART initiation; β_4_, BMI
