# Supplementary material for: Effect of antiretroviral therapy on longitudinal lung function trends in older children and adolescents with HIV-infection
Source: PLoS One. 2019 Mar 21;14(3):e0213556. doi: 10.1371/journal.pone.0213556 (PMC6428265; doi:10.1371/journal.pone.0213556)
Supplement: S4 Table — (DOCX) [file pone.0213556.s004.docx]

**S4 Table.** Likelihood ratio comparison of increasingly complex mixed-effects FEVz response models for the ART-naïve cohort.

|  | *Fixed effects parameters* | *Comparison* | *LogLikelihood* | *Likelihood ratio test* | *Df* | *p-value* |
| --- | --- | --- | --- | --- | --- | --- |
| **1** | β_0_ | - | -1036.4 | - | - | - |
| **2** | β_0_, β_1_ | 2 and 1 | -1034.8 | 3.2 | 1 | 0.074 |
| **3** | β_0_, β_2_ | 3 and 1 | -1033.5 | 5.8 | 1 | 0.015 |
| **4** | β_0_, β_2_, β_4_ | 4 and 3 | -1018.2 | 30.5 | 1 | 3.4 e^-08^ |

* All models have σ_u_^2^, σ_v_^2^_,_ σ_z_^2^, cov(U,V) as parameters. Parameters relate to; β_0_, intercept; β_1_, time on ART; β_2_, age at ART initiation; β_4_, BMI
