# Supplementary material for: Effect of antiretroviral therapy on longitudinal lung function trends in older children and adolescents with HIV-infection
Source: PLoS One. 2019 Mar 21;14(3):e0213556. doi: 10.1371/journal.pone.0213556 (PMC6428265; doi:10.1371/journal.pone.0213556)
Supplement: S7 Table — (DOCX) [file pone.0213556.s007.docx]

**S7 Table.** Likelihood ratio comparison of random-effects FEVz response models incorporating; **1**: Residual error, **2**: individual intercept, for the ART-established cohort.

|  | *Random effects* | *Comparison* | *LogLikelihood* | *Likelihood ratio test* | *p-value* |
| --- | --- | --- | --- | --- | --- |
| **1** | Z_ij_ | - | -496.9 | - | - |
| **2** | U_i_, Z_ij_ | 1 and 2 | -453.2 | 87.4 | < 2.2e^-16^ |
