# Supplementary material for: Effect of antiretroviral therapy on longitudinal lung function trends in older children and adolescents with HIV-infection
Source: PLoS One. 2019 Mar 21;14(3):e0213556. doi: 10.1371/journal.pone.0213556 (PMC6428265; doi:10.1371/journal.pone.0213556)
Supplement: S1 File — Questionnaire for ART-experienced cohort. (PDF) [file pone.0213556.s009.pdf]

# CLINICAL ASSESSMENT QUESTIONNAIRE FOR GHP & G1HN

| | | |
|---|---|---|
| **MODULE 1: Demographic Data** | | |
| **CA01** | Study No | [ ] |
| **CA02** | Date of Interview | [ ][ ] [ ][ ] [ ][ ][ ][ ] |
| **CA03** | Age | [ ][ ] |
| **CA04** | Date of Birth | [ ][ ] [ ][ ] [ ][ ][ ][ ] |
| **CA05** | Sex | Male [ ] Female [ ] |
| **CA06** | Are the Child's natural Parents alive | Both Parents alive [ ]<br>Mother alive, father dead / unknown [ ]<br>Father alive, mother dead [ ]<br>Both parents dead [ ]<br>Mother dead, father unknown [ ]<br>Mother unknown, father alive/dead [ ]<br>Don't know abut either parents [ ] |
| **CA07** | Is Participant index or siblings | Index [ ] Sibbling [ ] |
| **CA08** | Index Number | H[ ][ ][ ] |
| **Module 2:** | **HIV History** | |
| **CA09** | OI Clinic Number | [ ][ ][ ][ ][ ][ ][ ][ ][ ][ ][ ][ ] |
| **CA10** | Is the child aware of his/ her HIV status(mwana anoziva here paamire maererano neHIV | Yes [ ] No [ ] |
| **CA11** | Date of HIV diagnosis (DD/MM/YYYY) | [ ][ ] [ ][ ] [ ][ ][ ][ ] |
| **CA12** | Age at HIV diagnosis (years) | [ ][ ] |
| **CA13** | What was the main reason for the child to be tested (Chii chikonzero chakanyanyoita kuti mwana aende kunoongororwa ropa rake)? | Diagnosed with TB [ ]<br>Hospital Admission [ ]<br>Parent diagnosed/died of HIV [ ]<br>Repeated Illness ) [ ]<br>Often coughing [ ]<br>Pregnancy (PMTCT) or STI or sexual debut [ ]<br>As part of voluntary male circumcission [ ]<br>Short /not growing/puberty delay [ ]<br>Spontaneounsly offered by health care worker [ ]<br>Do not know [ ] |
| **C14** | What was the child's CD4 count on diagnosis | [ ][ ][ ][ ] cell/ul |

| | | |
|---|---|---|
| **CA15** | Please tick the current ARV drugs the child is taking and give date each drug was commenced: | AZT ( Zidovudine) ☐ ☐☐ ☐☐ ☐☐☐☐<br>D4T ☐ ☐☐ ☐☐ ☐☐☐☐<br>TDF (tenofovir) ☐ ☐☐ ☐☐ ☐☐☐☐<br>3TC/FTC ☐ ☐☐ ☐☐ ☐☐☐☐<br>DDI (didanosine) ☐ ☐☐ ☐☐ ☐☐☐☐<br>ATZ (atazanavir) ☐ ☐☐ ☐☐ ☐☐☐☐<br>LPV (Kaletra, alluvia) ☐ ☐☐ ☐☐ ☐☐☐☐<br>COTRIMOXAZOLE ☐ ☐☐ ☐☐ ☐☐☐☐<br>Nevirapine (NVP) ☐ ☐☐ ☐☐ ☐☐☐☐<br>Other ☐ ☐☐ ☐☐ ☐☐☐☐<br>specify ________ |
| **CA16** | What is the nurse's impression of the mode of HIV acquisition? | Mother to Child ☐<br>Blood transfusion or other parenteral ☐<br>Sexual transmission ☐ |
| **CA17** | Reason for this impression (specify) | ________ |
| **MODULE 3: Risk Factor History** | | |
| C**A18** | What is the main source of lighting in the child's home (taking into account frequency of powercuts munonyanya kushandisa chii pakuona usiku kumba kunogara mwana **(Choose only One)** | Electricity ☐<br>Candles ☐<br>Paraffin ☐<br>Dont Know ☐ |
| **CA19**. | What type of stove is most often used for cooking in the child's home (munonyanya kushandisa chitofu chakaita sei pakubika kumba kunogara mwana)? (most days, and if more than one, the alternatives must be used more than once per week.) | Open (wood) fire ☐<br>Gas Stove ☐<br>Electric Stove ☐<br>Paraffin Stove ☐<br>Sawdust stove ☐<br>Coal stove ☐<br>Gel Stove ☐<br>Other ☐<br>Don't Know ☐<br>specify ________ |
| **CA20** | Where exactly is the cooking done in the child's home (kubika uku kunonyatsoitikira pai kumba kunogara mwana) | Indoor Kitchen ☐<br>Kitchen in a separate house ☐<br>Kitchen outside the house but in a room ☐<br>Kitchen on the porch/ veranda outside the house ☐<br>Do not know ☐ |

**Child's Smoking History** (*These questions apply to children 11 years and over. Ask guardian first then child in the absence of guardian)*

Please ask the parent/guardian if he/she is comfortable with answering questions related to smoking. Any parents/guardians that are uncomfortable with these questions, can decline to answer

| | | |
|---|---|---|
| **CA21** | Does your child currently smoke cigarettes (parizvino mwana wenyu anoputa fodya here)? | Yes ☐ No ☐ N/A ☐ |
| **CA22** | Does your child drink alcohol (mwana wenyu anonwa doro here)? | Yes ☐ No ☐ N/A ☐ |
| **CA24** | Do you take snuff ? (Unoputa bute here) | Yes ☐ No ☐ N/A ☐ |
| **Family Smoking History** | | |
| **CA25** | Does anyone who lives in the child's home smoke cigarettes inside the house (pane mumwe munhu here anogara kumba kunogara mwana anoputa fodya)? | Yes ☐ No ☐ N/A ☐ |
| **CA26** | How many people smoke in the child's home (Kune vanhu vangani vanoputa fodya kumba kunogara mwana)? If none please put "00" 99 for don't know or don't want to answer | ☐☐ |
| **Living and Working Conditions** | | |
| **CA27** | Does the child have contact or play with any of the following animals at least once a week (mwana anombogumana kana kutamba nechero mhuka pane dzinotevera) (**CHECK ALL THAT APPLY)?** | |
| | 1. Dogs (imbwa) | Yes ☐ No ☐ N/A ☐ |
| | 2. Cats (katsi) | Yes ☐ No ☐ N/A ☐ |
| | 3. Birds and poultry (huku kana shiri) | Yes ☐ No ☐ N/A ☐ |
| | 4. Goats or cows (mombe kana mbudzi) | Yes ☐ No ☐ N/A ☐ |
| **CA28** | Has the child had exposure to any of the following in the last 2 years (mwana akamboendwa nezvinotevera here mumakore maviri apfuura)? | |
| | 1. Dust (huruva) (e.g., wood dust, agricultural dust, sawdust) | Yes ☐ No ☐ N/A ☐ |
| | 2. Smoke (hutsi) (e.g., trash burning) | Yes ☐ No ☐ N/A ☐ |
| | 3. Chemical fumes (munhuwi wemishonga) (e.g., glue, paint) | Yes ☐ No ☐ N/A ☐ |
| **CA29** | Has the child done any of the following (mwana akamboita zvinotevera here)? | |
| | 1.Mining (kuchera matombo) | Yes ☐ No ☐ N/A ☐ |
| | 2. Farming (kurima) | Yes ☐ No ☐ N/A ☐ |
| | 3. Chemical manufacturing (kugadzira mishonga) | Yes ☐ No ☐ N/A ☐ |
| | 4. Welding (kugadzira simbi) | Yes ☐ No ☐ N/A ☐ |

| | | |
|---|---|---|
| **CA30** | What is the child's main source of transportation (mwana anonyanya shandisa chii pakufambisa) (CHOOSE ONLY ONE, SARUDZAI CHIMWE)? | Walking ☐<br>Car ☐<br>Commuter Omnibus or Bus ☐<br>Motorcycle ☐<br>Bicycle ☐<br>Don't Know ☐ |
| **CA31** | How many months has the child lived in the following settings in the last two years(mwana akagara munzvimbo idzi kwenguva yakareba sei mumakore maviri apfuura)? (ADD UP TO 24) | Urban ☐☐ months<br>Rural ☐☐ months<br>Don't know ☐☐ months |
| **CA32** | How has the drinking water been treated in the home (mvura yamunonwa kumba munoichenesa sei)? | Never ☐<br>Boiling ☐<br>Chlorine (Water guard) ☐<br>Filter ☐<br>Unknown ☐ |
| **MODULE 4: Clinical History** | | |
| **CA33**. | How many times has the child been admitted to the hospital in the last 12 months (mwana akambogara muchipatara kangani mugore rapfuura? (00=never; 99=don't know) | ☐☐ |
| **CA34** | How many times has the child been admitted to the hospital for a chest infection/pneumonia in the last 12 months( mwana akambogara kangani muchipata pamusoro pekurwara nechipfuva mugore rapfura)? (00=never; 99=don't know) | ☐☐ |
| **CA35** | How many times has the child been given antibiotics for a chest infection/pneumonia in the last 12 months (include hospital admissions), (mwana akapihwa kangani mishonga inorapa chipfuva mugore rapfuura kusanganisira paakambogara muchipatara)? (00=never; 99=don't know) | ☐☐ |
| **CA36** | Has the child ever been diagnosed with any of the following(check notes): | |
| | Measles | Yes ☐ No ☐ Don't Know ☐ |
| | PCP | Yes ☐ No ☐ Don't Know ☐ |
| | Asthma | Yes ☐ No ☐ Don't Know ☐ |
| | Other problem or weakness of the lungs (kumwe kurwara kwemapapu | Yes ☐ No ☐ Don't Know ☐ |
| | Heart problem (chirwere chemoyo) | Yes ☐ No ☐ Don't Know ☐ |
| | High blood pressure | Yes ☐ No ☐ Don't Know ☐ |
| | Murmur of the heart (kurira kwemoyo) | Yes ☐ No ☐ Don't Know ☐ |
| | Rheumatic fever | Yes ☐ No ☐ Don't Know ☐ |
| | Has the child ever been advised not to do sports by a doctor | Yes ☐ No ☐ Don't Know ☐ |
| | Has anyone in the family died at the age of 40 of a heart problem | Yes ☐ No ☐ Don't Know ☐ |
| | Has anyone in the family have diabetes | Yes ☐ No ☐ Don't Know ☐ |

Ver 1.0 Aug 2014 **CLINICAL ASSESSMENT QUESTIONNAIRE FOR GHP & G1HN**

| | | |
|---|---|---|
| **CA37** | Has the child had any of the following vaccines (CHECK ALL THAT APPLY)?<br>Measles vaccine | Yes ☐ No ☐ Don't Know ☐ |
| | BCG (TB) vaccine | Yes ☐ No ☐ Don't Know ☐ |
| | Pentavalent vaccine (DTP, haemophilus, Hep B) | Yes ☐ No ☐ Don't Know ☐ |
| | Pneumococcal conjugate vaccine (PCV) | Yes ☐ No ☐ Don't Know ☐ |
| **CA38** | From your knowledge, has the child received all their childhood vaccinations (sokuziva kwenyu mwana akadzivirirwa here pazvirwere zvese zvehudiki)? | Yes ☐ No ☐ Don't Know ☐ |
| **CA39** | Is the child currently on TB treatment (Parizvino mwana ari kurapwa here TB)? | Yes ☐ No ☐ Don't Know ☐ |
| **CA40** | Has the child ever been treated for TB in the past excluding the current episode (mwana akamborapwa here TB kusanganisira iye zvino)? | Yes ☐ No ☐ Don't Know ☐ |
| **CA41** | How many times has the child been treated for TB (excluding the current episode)? Code 9 if N/A (i.e. never had TB) | ☐☐ |
| **MODULE 5: SYMPTOM HISTORY** | | |
| **TB Screening**<br>**CA42** | In the last 4 weeks, has the child been sweating at night (drenchingnight sweats: beddings wet with sweat) mumasvondo mana apfuura mwana aimbodikitira husiku here? | Yes ☐ No ☐ |
| **CA43** | In the last 4 weeks, did the child have any fevers/body feeling hot (mumasvondo mana apfuura mwana akambopisa muviri here)? | Yes ☐ No ☐ |
| **CA44** | In the last 4 weeks, has the child been losing weight (mumasvondo mana apfuura mwana akambodzikira muviri wake here)? | Yes ☐ No ☐ |
| **CA45** | In the last 12 months, has the child ever stayed in the same house/household with someone with TB (mugore rapfuura mwana akambogara mumba mumwe chete nemunhu ane TB here)? | Yes ☐ No ☐ |
| **Breathless**<br>**CA46** | Is the child currently breathless (parizvino mwana anombozarirwa here)? not necessarily immediately, but over the last weeks) | Yes ☐ No ☐ |
| **CA47** | When did the child first start feeling breathless (mwana akatanga rinhi kuzarirwa)? | Less than 1 Month Ago ☐<br>1-3 months ago ☐<br>More than 3 months ago ☐<br>Don't know ☐ |
| **CA48** | How would you describe the pattern of the child's breathlessness (mungatsanangure sei mazarirwe anoita mwana wenyu)? | Comes and goes with normal peiods in between ☐<br>Comes and goes but not recovering to normal state ☐<br>Progressively getting worse ☐<br>Don't Know ☐ |

| | | |
|---|---|---|
| **CA49** | Does the child get breathless when dressing or is too breathless to leave the house (mwana anombozarirwa here kana achipfeka kana kutadza kubuda mumba)? | Yes ☐ No ☐ |
| **CA50** | Does the child have to stop for breath after walking 100m (mwana anombomira here kana achifamba kuti ambofema)? | Yes ☐ No ☐ |
| **CA51** | Does the child walk slower than most people or has to stop after 15min (Mwana anombofamba kumashure kwevamwe vana here kana kumbomira kwapera maminitsi makumi mashanu)? | Yes ☐ No ☐ |
| **CA52** | Is the child short of breath when hurrying on the level or walking uphill (mwana anozarirwa here kana achikwira makata)? | Yes ☐ No ☐ |
| **CA53** | Does the child get breathless on moderate exercise (if breathless on strenuous exercise, code NO) (Mwana anozarirwa kana abva kuita basa rakaomarara)? | Yes ☐ No ☐ |
| **CA54** | Has the child been coughing most days for more than a month (mwana akakosora zvakanyanya herezvekudarika mwedzi)? | Yes ☐ No ☐ Don't Know ☐ |
| **CA55** | What is the frequency of the child's cough (mwana anokosora zvakanyanya sei)? | Cough less than 5 cycles per day ☐<br>Cough more than 10 cycles per day ☐<br>Distressing cough most of the day ☐<br>Not coughing for more than a month or do not know ☐ |
| **CA56** | Has the cough changed over time. If child coughs regularly anyway(kukosora kwake kwasanduka zvakadii pamwedzi mitanhatu yapfuura)? Kana achikosora zvakanyanya | Same ☐<br>Improving ☐<br>Getting worse ☐<br>Not coughing for more than a month or do not know ☐ |
| **CA57** | Does the child cough up sputum (mwana anokosora makararwa here)? | Yes ☐<br>No ☐<br>Not coughing for more than a month don't Know ☐ |
| **CA58** | How much sputum does the child bring up (mwana anokosora makararwa akawanda zvakadii)? | Less than a spoon ☐<br>A few table spoon ☐<br>A Cup ☐<br>Not coughing more than a month or do not know ☐ |
| **CA59** | Does the child have a history of frequently choking on food or water (mwana anombodzipwa nechikafu here)? | Yes ☐ No ☐ |

**Wheeze and Asthma**

| | | |
|---|---|---|
| **CA60** | LYWH Have you had wheezing or whistling in the chest in the last 6 months (wakambotadza kufema here kana kurira muchipfuva mumwedzi mitanhatu yapfuura)? | Yes ☐ No ☐ |
| **CA61** | In the last 12 months, has wheezing ever been severe enough to limit your speech to only one or two words at a time between breaths (mugore rapfura kutadza kufema kwakambonyanya zvekutadzisa kutaura | Yes ☐ No ☐ N/A ☐ |
| **CA62** | Do you use an inhaler (Unoshandisa inhaler here)?<br>How often do you use an inhaler (unoshandisa inhaler kangani)? | Once a week ☐ > once a week ☐<br>Daily ☐ N/A ☐ |

## CLINICAL ASSESSMENT QUESTIONNAIRE FOR GHP & G1HN

| | | |
|---|---|---|
| **CA63** | Do you use salbutamol tablets (unoshandisa mapiritsi anonzi salbutamol)? | Yes ☐ No ☐ N/A ☐ |
| **CA64** | Have you had any ankle swelling in the past 3 months (wakambozvimba makumbo mumwedzi mitatu yapfuura)? | Yes ☐ No ☐ |
| **CA65** | Have you had any chest pains during or after exercise in the past 3 months (unomborwadziwa nechipfuva pakushanda or wapedza kushanda zvakanyanya mumwedzi mitatu yapfura)? | Yes ☐ No ☐ |
| **CA66** | Have you had chest pains at rest in the past 3 months (wanga uchimborwadziwa muchipfuva here pakuzorora)? | Yes ☐ No ☐ |
| **CA67** | Does your heart ever beat rapidly for no apparent reason (moyo wako unomborova here pasina zvawaita)? | Yes ☐ No ☐ |
| **CA68** | Have you ever fainted in the past 12 months (wakambofenda mugore rapfura) | Yes ☐ No ☐ |
| **CA69** | Have you ever had any dizzy spells in the past 12 months (wakambonzwa dzungu here mugore rapfuura) | Yes ☐ No ☐ |
| **CA70** | Has the child ever broken a bone in their lifetime? This includes all fractures, cracks, chips and breaks. | Yes ☐ No ☐ |

| **CA71** | Which bone did the child break? | **Total no of times broken same area on same side** | **Other Side** | **No of times bones broken during a serious accident, fall of more than 3 metres being hit by a heavy moving object** |
|---|---|---|---|---|
| | Fingers and /Toes | ☐ | ☐ | ☐ |
| | Bone in arm/shoulder | ☐ | ☐ | ☐ |
| | Bone in leg | ☐ | ☐ | ☐ |
| | Spine (back) | ☐ | ☐ | ☐ |
| | Other bones | ☐ | ☐ | ☐ |

| **CA72** | How were the bones managed (tick all that apply | | | | |
|---|---|---|---|---|---|
| | Fingers and/or Toes | X-Ray ☐ | Surgery or Operation ☐ | Cast or Splint ☐ | Nothing ☐ |
| | Bone in arm/shoulder | X-Ray ☐ | Surgery or Operation ☐ | Cast or Splint ☐ | Nothing ☐ |
| | Bone in leg | X-Ray ☐ | Surgery or Operation ☐ | Cast or Splint ☐ | Nothing ☐ |
| | Spine (back) | X-Ray ☐ | Surgery or Operation ☐ | Cast or Splint ☐ | Nothing ☐ |
| | Other bones | X-Ray ☐ | Surgery or Operation ☐ | Cast or Splint ☐ | Nothing ☐ |
