# Supplementary material for: Effect of antiretroviral therapy on longitudinal lung function trends in older children and adolescents with HIV-infection
Source: PLoS One. 2019 Mar 21;14(3):e0213556. doi: 10.1371/journal.pone.0213556 (PMC6428265; doi:10.1371/journal.pone.0213556)
Supplement: S2 File — Questionnaire for ART-naïve cohort. (PDF) [file pone.0213556.s010.pdf]

5926539100

ZT05 ZENITH INITIAL ASSESSMENT FORM

**A01**	STUDYNO	STUDY NUMBER	Z
**A02**	DATE	Date of assessment (dd/mm/yyyy)	/ / 2 0 1
**A03**	INTID	Interviewer ID	
**A04**	SURN	Surname	
**A05**	FIRST	First Name	
**A06**	DOB	Date of Birth (dd/mm/yyyy)	
**A07**	AGE	Age at last birthday	
**A08**	SEX	Gender	Male ☐ Female ☐
**A09**	KEYIN	At this visit, who is providing information regarding the child? (check all that apply)	Child (patient) ☐ Sibling ☐ Mother ☐ Other ☐ Father ☐ specify ______
**A10**	CGUARD	Current main caregiver (use Relationship key below)	☐
**A11**	GUARDC	Has child had a different caregiver (other than parent) since birth (consider the biological parents as the caregiver 1 at birth)<br>*If A11 answer is N0 skip to A28*	Yes ☐ No ☐

CAREGIVER HISTORY

Earliest to current if had > 1 caregiver from birth

if caregiver changed from biological parent (at birth) to someone else, even if subsequently returned to parent

Use this key to answer A10, A12, A16, A20 & A24

Relationship Key

1 = Father and/or Mother	4 = Aunt and/ or Uncle	7 = Nephew or niece
2 = Sibling	5 = Father and/or Stepmother	8 = Institution
3 = Grandmother and/or Grandfather	6 = Mother and/or Stepfather	9 = No caregiver

**A12**	RELG1	Relationship of Caregiver **2** to participant	☐
**A13**	YEARG1	When changed to Caregiver **2** (mm/yyyy)	/
**A14**	RESG1	Reason for change to Caregiver **2**	______
**A15**	LOCG1	Where living with Caregiver **2**	Harare ☐ Out of Harare ☐
**A16**	RELG2	Relationship of Caregiver **3** to participant	☐
**A17**	YEARG2	When changed to Caregiver **3** (mm/yyyy)	/
**A18**	RESG2	Reason for change to Caregiver **3**	______
**A19**	LOCG2	Where living with Caregiver **3**	Harare ☐ Out of Harare ☐
**A20**	RELG3	Relationship of Caregiver **4** to participant	☐

**A21**	YEARG3	When changed to Caregiver **4** (mm/yyyy)	☐☐/☐☐☐☐
**A22**	RESG3	Reason for change to Caregiver **4**	________
**A23**	LOCG3	Where living with Caregiver **4**	Harare ☐ Out of Harare ☐
**A24**	RELG4	Relationship of Caregiver **5** to participant	☐
**A25**	YEARG4	When changed to Caregiver **5** (mm/yyyy)	☐☐/☐☐☐☐
**A26**	RESG4	Reason for change to Caregiver **5**	________
**A27**	LOCG4	Where living with Caregiver **5**	Harare ☐ Out of Harare ☐
**HIV TESTING HISTORY**			
**A28**	FIRST	Was the HIV test in this clinic the clild's first HIV test	Yes ☐ No ☐ Don't know ☐
**A29**	FIRSTY	If **Yes** to **A28**, was the child ever offered a test before by a Health Care Worker?	Yes ☐ No ☐
**A30**	FIRSTN	If answer **"No"** to **A28**, date of previous HIV test(mm/yy)	☐☐/☐☐☐☐
**A31**	RES	If answer "No" to **A28**, result of the previous HIV test:	Positive ☐ Negative ☐
**A32**	SUSP	Did you suspect the child might be HIV-positive before this test?	Yes ☐ No ☐
**FAMILY HISTORY**<br>**Ask about all Natural siblings i.e sharing same mother**			
**A33**	SIB	How many natural siblings does the child have who are alive	(Enter "99" if unknown) ☐☐
**A34**	SIBD	How many natural siblings does the child have who are dead	(Enter "99" if unknown) ☐☐
**A35**	SIBH	How many natural siblings does the child have who are /were HIV+ve	(Enter "99" if unknown) ☐☐
**A36**	SIBA	How many natural siblings does the child have who are taking ART	(Enter "99" if unknown) ☐☐
**A37**	MOA	Is natural mother alive	Yes ☐ No ☐ Don't know ☐
**A38**	MOH	Is/was natural mother HIV +ve	Yes ☐ No ☐ Don't know ☐
**A39**	MOA	Is/was natural mother taking ART	Yes ☐ No ☐ Don't know ☐
**A40**	FAA	Is natural father alive	Yes ☐ No ☐ Don't know ☐
**A41**	FAH	Is/was natural father HIV+ve	Yes ☐ No ☐ Don't know ☐
**A42**	FAA	Is/was natural mother taking ART	Yes ☐ No ☐ Don't know ☐

**A43**	HSIZE	No of people in Household	☐☐
**A44**	FUEL	What are the types of fuel are commonly (at least once a week) used in the household? (check all that apply)	Firewood (indoors) ☐<br>Firewood (outdoors) ☐<br>Paraffin ☐<br>Electricity ☐<br>Gas ☐<br>Charcoal (indoors) ☐<br>Crop residue ☐<br>Charcoal (outdoors) ☐
**A45**	SMOKE	How many people in the household smoke cigarettes	☐☐
**A46**	HHTB	How many people in the household had TB in past 12 months (put 00 if none)	☐☐
**Answer next three questions if one or more persons in household had TB in past year**			
**A47**	CONTB	Did child have close contact when they had TB? (check yes if close contact - sleeping in same room/or hugging - with at least 1 person who had TB)	Yes ☐ No ☐
**A48**	CGHTB	Did the person(s) cought when they had TB? (Tick yes if at least one contact had cough)	Yes ☐ No ☐
**A49**	MONTB	For how many months did the person(s) who had TB in the household get TB treatment	Contact 1: ☐☐ months<br>Contact 2: ☐☐ months<br>Contact 3: ☐☐ months
**SOCIAL HISTORY**			
**A50**	SCHA	Is the child currently school	Yes ☐ No ☐ Never attended ☐
**If not attending school:**			
**A51**	SCHYR	When did child stop going to school? (YYYY)	☐☐☐☐
**A52**	SCHTRN	Attending any other non-school training?	Yes ☐ No ☐
**If attending school:**			
**A53**	SCHG	Current Level of education	Form ☐ Grade ☐
**A54**	SCHMS	No. of school days missed in last 3 months	☐☐

**A55**	SCHRES	Main reason for missing school (if missed more than 5 consecutive days of school	Illness ☐<br>No school fees ☐<br>Relocation/change of caregiver ☐<br>Death in family ☐<br>Religious reasons ☐<br>Teachers strike / security reasons ☐<br>Taking care of househol member ☐<br>Not wanting to go to school ☐
**A56**	DISCL	Does the child know that he/she has HIV?	Yes ☐ No ☐
**A57**	DISCLN	If child does not know that he/she has HIV, what is the reason for him/her not knowing (check all that apply	Child is too young ☐<br>Child doesn't understand ☐<br>Child might tell others ☐<br>It will hurt child to know ☐<br>I don't know how to tell the child ☐<br>The nurse or counsellor should tell child ☐<br>Another relative should disclose ☐<br>Child is not sick ☐<br>Other ☐<br>Specify ________
**Does the child know the HIV status of their:**			
**A58**	KSTATM	Mother	Yes ☐ No ☐
**A59**	KSTATF	Father	Yes ☐ No ☐
**A60**	KSTATC	Caregiver (if applicable)	Yes ☐ No ☐
**A60a**	KSIB	Sibling	Yes ☐ No ☐
**Who have been told HIVstatus of child:**			
**A61**	DISCLF	Father	Yes ☐ No ☐ N/A ☐
**A62**	DISCLM	Mother	Yes ☐ No ☐ N/A ☐
**A63**	DISCLS	Siblings	Yes ☐ No ☐ N/A ☐
**A64**	DISCLR	Relatives	Yes ☐ No ☐
**A65**	DISCLP	Friends	Yes ☐ No ☐
**A66**	DISCLC	Church pastor	Yes ☐ No ☐ N/A ☐
**A67**	DISCLT	School class teacher	Yes ☐ No ☐ N/A ☐

**A68**	DISLHT	School head teacher	Yes ☐ No ☐ N/A ☐
**A69**	DISCLO	Other (Specify)	______________________
		**CLINICAL HISTORY**	
**A70**	PMTCT	Did mother receive ART for PMTCT?	Yes ☐ No ☐ Don't know ☐
**A71**	BCG	Did the child receive a BCG vaccine?	Yes ☐ No ☐ Don't know ☐
**A72**	TB	Has child previously been treated for Tuberculosis? (excluding current TB)	Yes ☐ No ☐ Don't know ☐
**A73**	DTETB	How many times has child been treated for TB?(excluding current TB)	☐☐
**A74**	TREATTB	When was child last treated for TB? (yyyy)(excluding current TB)	☐☐☐☐
**A75**	TMONTB	For how many months was the child on TB tratment during your last episode of TB (excluding current TB)	☐☐
**A76**	ADMIT	Has the child been admitted to hospital in the past? (stay in hospital for more than 1 night)	Yes ☐ No ☐ Don't know ☐
**A77**	HOSPC	How many times has the child been admitted to hospital since birth?	☐☐
**A78**	CLINICC	How many times has the child gone to a clinic in the past six months?	☐☐
**A79**	MED	Has child previously taken Antiretroviral medication?	Yes, but only for PMTCT ☐<br>Yes, previously treated with ART ☐<br>No ☐<br>Don't know ☐
**A80**	TRANS	Nurse's impression of likely mode of HIV transmission?	MTCT ☐ Parenteral ☐ Horizontal ☐
**A81**	PTRANS	Reason for impression	______________________
		**HEALTH OF THE CHILD**	
**Is the child currently taking any of the following:**			
**A82**	MTB	TB treatment	Yes ☐ No ☐
**A83**	MCOT	Cotrimoxazole	Yes ☐ No ☐
**A84**	MANT	Other Antibiotics	Yes ☐ No ☐
**A85**	MOTH	Other (prescription/ non-prescription/herbal/traditional	Yes ☐ No ☐

Within the last month, has the child experienced any of the following symptoms

**A86**	CGH	Cough	Yes ☐ No ☐ Don't know ☐
**A87**	WGHTL	Weight loss	Yes ☐ No ☐ Don't know ☐
**A88**	FEVR	Hot body or fever	Yes ☐ No ☐ Don't know ☐
**A89**	NSWT	Night sweats (has to change clothes/bedsheets/need towel to dry self)	Yes ☐ No ☐ Don't know ☐
**A90**	BRTH	Difficulty breathing	Yes ☐ No ☐ Don't know ☐
**A91**	FATG	Fatigue	Yes ☐ No ☐ Don't know ☐
**A92**	DIAR	Diarrhoea	Yes ☐ No ☐ Don't know ☐
**A93**	HCHE	Persistent Headaches	Yes ☐ No ☐ Don't know ☐
**A94**	APPE	Poor appetite	Yes ☐ No ☐ Don't know ☐
**A95**	NAUS	Nausea/Vomiting	Yes ☐ No ☐ Don't know ☐
**A96**	RASH	Rash	Yes ☐ No ☐ Don't know ☐
**A97**	MEM	Trouble remembering	Yes ☐ No ☐ Don't know ☐
**A98**	THRSH	Thrush in mouth	Yes ☐ No ☐ Don't know ☐
**A99**	SWAL	Difficulty/Pain on swallowing	Yes ☐ No ☐ Don't know ☐
**A100**	STI	STI symptoms	Yes ☐ No ☐ Don't know ☐
**A101**	HLTH	How does the caregiver/ child rate ther child's general health over the past 3 months?	Excellent ☐ Good ☐ Fair ☐ Poor ☐
**A102**	STAT	Current functional status	Normal ☐ Able to perform usual activities (work/study/housework) and self-care (wash/eat/dress)<br>Ambulatory ☐ Cannot perform usual activities but can self-care without assistance<br>Bedridden ☐ Cannot self care without assistance

Does the child have any of these problems

**A103**	EAR	Hearing problems	Yes ☐ No ☐
**A104**	EYE	Visual problems (other than refractive error)	Yes ☐ No ☐
**A105**	MUSC	Musculoskeletal problems	Yes ☐ No ☐

**A106**	SPCH	Speech problems	Yes ☐ No ☐
**WHO Clinical stage** (include current symptoms or symptoms with documented history only			
**Stage 1 or 2**			
**A107**	ASY	Asymptomatic	Yes ☐ No ☐
**A108**	LMPH	Persistent generalised lymphadenopathy	Yes ☐ No ☐
**A109**	ERUP	Papular pruritic eruption	Yes ☐ No ☐
**A110**	HERP	Herpes zoster (shingles)	Yes ☐ No ☐
**A111**	ENT	Recurrent or chronic URTI (ear/throat/sinus infections)	Yes ☐ No ☐
**A112**	WART	Extensive wart virus infection	Yes ☐ No ☐
**A113**	MOLL	Molluscum contagiosum	Yes ☐ No ☐
**A114**	FUNG	Fungal nail infection	Yes ☐ No ☐
**Stage 3 or 4**			
**A115**	GING	Gingivitis/periodontitis	Yes ☐ No ☐
**A116**	THRSH	Thrush in the mouth	Yes ☐ No ☐
**A117**	STHSH	Thrush with pain on swallowing	Yes ☐ No ☐
**A118**	DIAR	Chronic Diarrhoea (for > 2 weeks)	Yes ☐ No ☐
**A119**	FEVR	Fever >1 month	Yes ☐ No ☐
**A120**	STNT	Severe stunting (<3rd centile) **refer to growth chart**	Yes ☐ No ☐
**A121**	WAST	Wasting (<3rd centile) **refer to growth chart**	Yes ☐ No ☐
**A122**	PNEU	Pneumonia or other serious bacterial infection (e.g. meningitis/bone/joint infection)	Yes ☐ No ☐
**A123**	TB	Tiberculosis (PTB or EPTB) in past two years	Yes ☐ No ☐
**A124**	HIVEN	HIV encephalopathy Not met milestones (Speech, Motor, Social) OR memory problems	Yes ☐ No ☐
**A125**	KP	Kaposi Sarcoma	Yes ☐ No ☐
**A126**	MENG	Cryptococcal meningitis	Yes ☐ No ☐
**A127**	LYMP	Lymphoma	Yes ☐ No ☐
**A128**	WHO	WHO Stage	Stage I or II ☐ Stage III or IV ☐

CLINICAL EXAMINATION			
A129	SCAR	BCG Scar seen	Yes ☐ No ☐
A130	CLUB	Finger clubbing	Yes ☐ No ☐
A131	KS	Kaposi Sarcoma lesions (mouth or skin)	Yes ☐ No ☐
A132	PRU	Papular pruritic eruption	Yes ☐ No ☐
A133	COTS	Cotrimoxazole skin rash<br>**If yes stop cotrimoxazole**	Yes ☐ No ☐
A134	PWART	Planar warts	Yes ☐ No ☐
A135	VWART	Verrucous warts	Yes ☐ No ☐
A136	MOLL	Molluscum contagiosum	Yes ☐ No ☐
A137	FUNG	Fungal nail infection	Yes ☐ No ☐
A138	THR	Oral Thrush	Yes ☐ No ☐
A139	GING	Gingivitis/ periodontitis	Yes ☐ No ☐
A140	TEMP	Temperature	☐ ☐ ☐.☐ oC
A141	HGTST	Standing Height	☐ ☐ ☐.☐ cm
A142	HGTSI	Sitting Height (only measure if child cannot stand)	☐ ☐ ☐.☐ cm
A143	WGHT	Weight	☐ ☐.☐ kg

PLAN			
A144	PCD4	CD4 sample taken	Yes ☐ No ☐
A145	BSTOR	Blood store sample taken	Yes ☐ No ☐
A146	REFDTB	Referred for TB tests to BRIDH<br>(If TB symptoms & not taking TB drugs)<br>**Provide client with Forms PD01 and PD02**	Yes ☐ No ☐
A147	PGRW	Growth form (ZT08) completed	Yes ☐ No ☐
A148	PLUNG	Lung function & spirometry booked/done	Yes ☐ No ☐
A149	PGCHT	Growth chart plotted	Yes ☐ No ☐
A150	POTH	Any other treatment given	Yes ☐ No ☐
A151	PSPEC	Specify the treatment given	________________
A152	DAPPT	Patient's next appointment (2weeks)<br>(dd/mm/yyyy)	☐☐/☐☐/2 0 1 ☐
